# Potential for Pancreatic Maturation of Differentiating Human Embryonic Stem Cells Is Sensitive to the Specific Pathway of Definitive Endoderm Commitment

**DOI:** 10.1371/journal.pone.0094307

**Published:** 2014-04-17

**Authors:** Maria Jaramillo, Shibin Mathew, Keith Task, Sierra Barner, Ipsita Banerjee

**Affiliations:** 1 Department of Bioengineering, University of Pittsburgh, Pittsburgh, Pennsylvania, United States of America; 2 Department of Chemical and Petroleum Engineering, University of Pittsburgh, Pittsburgh, Pennsylvania, United States of America; 3 McGowan Institute for Regenerative Medicine, University of Pittsburgh, Pittsburgh, Pennsylvania, United States of America; University of Newcastle upon Tyne, United Kingdom

## Abstract

This study provides a detailed experimental and mathematical analysis of the impact of the initial pathway of definitive endoderm (DE) induction on later stages of pancreatic maturation. Human embryonic stem cells (hESCs) were induced to insulin-producing cells following a directed-differentiation approach. DE was induced following four alternative pathway modulations. DE derivatives obtained from these alternate pathways were subjected to pancreatic progenitor (PP) induction and maturation and analyzed at each stage. Results indicate that late stage maturation is influenced by the initial pathway of DE commitment. Detailed quantitative analysis revealed WNT3A and FGF2 induced DE cells showed highest expression of insulin, are closely aligned in gene expression patterning and have a closer resemblance to pancreatic organogenesis. Conversely, BMP4 at DE induction gave most divergent differentiation dynamics with lowest insulin upregulation, but highest glucagon upregulation. Additionally, we have concluded that early analysis of PP markers is indicative of its potential for pancreatic maturation.

## Introduction

Diabetes affects over 20 million people in the US [1]. In diabetic patients the body is unable to produce or properly use insulin. The most common treatment for type I diabetes consists of exogenous insulin supply. Other treatment alternatives include transplantation of cadaveric pancreas or isolated pancreatic islets [2], but the main limitations remain in the lack of available donor tissue. Human embryonic stem cells (hESCs) have been suggested as an alternative transplantable cell source for treatment of diabetes [3]. However, exploitation of the full potential of hESCs requires a robust protocol for generation of mature and functional cell types. Pancreatic differentiation of hESCs has received considerable attention over the last decade. While there has been some success in deriving insulin (*INS*) positive cells from hESCs, typically the differentiated cells are limited in yield and functionality [4]. Most differentiation protocols involve a stage-wise directed differentiation strategy that mimics stages of pancreatic organogenesis by modulating pathways known to be involved in pancreatic development [3]. The first critical stage of pancreatic differentiation is the commitment to definitive endoderm (DE). Studies over the last decade have established multiple alternate pathways for DE induction of hESCs. While all of these alternate routes yield efficient DE, it is not obvious how sensitive pancreatic maturation will be to such early pathways of DE induction. Thus, the method of DE induction remains somewhat arbitrary, being assessed only by the presence of DE markers and not by its potential for pancreatic maturation.

In this work we are addressing this issue by evaluating the sensitivity of late stage pancreatic maturation on initial pathways of DE induction. We induced DE differentiation of hESCs by activation of the Nodal pathway through Activin A, in combination with modulation of one of the following pathways: WNT, BMP, PI3K and FGF. All of these pathways have been identified as key players at multiple stages of pancreatic development. Activin A, a TGF-β family protein, has been long identified to mimic nodal, which results in mesoderm and DE formation [5]. FGF plays critical roles in several stages of pancreatic development. In the ventral pancreatic endoderm, FGF signaling comes from the adjacent endothelial mesoderm and at high concentrations specifies hepatic development at the expense of pancreatic differentiation [6]. Conversely, in the dorsal pancreatic endoderm, FGF signaling comes from the notochord and works as a sonic hedgehog (SHH) inhibitor, therefore inducing expression of *PDX1* and further pancreatic development [6]. Additionally, BMP4 signaling from the septum transversum acts synergistically with FGF2 to induce hepatic differentiation at the expense of ventral pancreas development [7]. However, BMP4 signaling has been found to act synergistically with Activin and FGF2 to promote mesendoderm differentiation in human pluripotent stem cells [8] and has been used in combination with Activin for DE induction in pancreatic differentiation studies [9]–[11]. Similarly, inhibition of WNT signaling by proximal mesoderm has been implicated in proper pancreatic and hepatic progression from the foregut [7],while activation of WNT induces mesendoderm formation in pluripotent stem cells from mouse and human sources [12]–[14]. Lastly, PI3K was first reported as a negative regulator of cellular differentiation, and its inhibition has more recently been linked to proper endoderm formation under high nodal signaling conditions [15]. Studies have also linked PI3K suppression at later stages with proper endocrine specification [16].

Due to the high complexity of these pathways and their role in pancreatic progression, a more thorough analysis of their effects is needed. The aim of this study is to compare previously identified pathways of DE induction, analyze their pancreatic potential, compare differentiation of these derivatives with existing reports on *in vivo* pancreatic organogenesis and identify markers that can be useful indicators of pancreatic differentiation at early stages of the differentiation program.

## Materials and Methods

### hESC Maintenance

H1 hESCs (WiCell) were maintained in feeder free conditions as previously described [17].

### Pancreatic Differentiation Protocol

Once hESCs reached an average colony size of 1 mm in diameter, DE induction media was added for 4 days with media change every day. After 4 days media was replaced with pancreatic progenitor (PP) media for 2 days with media change every day. After 2 days, all-Trans Retinoic acid was added to the PP media for 2 additional days with media change every day. Media was then replaced with maturation media. After 2 days DAPT was added to maturation media. Cells were maintained in this media for 1 week with media change every day. Media formulations are found in table S1.

### Proliferation and Cell Death Quantification

On day 0 of the protocol, several wells were treated with Accutase and starting cell density was estimated using a hemocytometer. 24 hours after initial DE media exposure, cell death was quantified by counting floating cells in the media and normalized with respect to the starting cell density. Additionally, the remaining attached cells were harvested with Accutase, stained with propidium iodide in PBS at a concentration of 10 ug/ml and the number of dead cells (PI positive) was quantified by flow cytometry. For quantification of cell number throughout the entire protocol, cells were exposed to alamar blue at day 0 according to manufacturer's instructions for quantification of cell number. This procedure was repeated at the end of each stage of differentiation (days 4,8,15), and cell number was calculated as described in the product manual, using day 0 values as a control for each of the stages.

### Quantitative Polymerase Chain Reaction

qPCR was performed as previously described [11]. A list of the primers used can be found in the table S2. ΔCt values were calculated by subtracting the respective Ct value for GAPDH from the Ct value of the marker(s) of interest. ΔΔCt values were calculating by subtracting the ΔCt values for undifferentiated cells for the marker of interest from the ΔCt value for the same marker in each group. Relative expression was found by calculating 2^−ΔΔCt^.

### Flow Cyotometry

Flow cytometry was performed as previously described [11]. As a control for non-specific staining, cells were incubated in secondary antibody only. Cells were analyzed using an Accuri C6 flow cytometer. Antibodies and concentrations can be found in the table S3. For cell cycle analysis, cells were harvested and dissociated with Accutase, rinsed, centrifuged and resuspended in ice-cold 70% ethanol and fixed overnight in −20°C. Cells were rinsed and suspended in DNA staining buffer (PBS+0.1% Triton-X+0.2 mg/mL DNAse-free RNAse+0.01 mg/mL/1 million cells propidum iodide) for 25 minutes at RT. Stained cells were then directly analyzed on an Accuri C6 flow cytometer, the output of which being a histogram of the DNA content for the cellular population in each sample. To accurately determine the fractions of the cellular population in each phase of the cell cycle from these data, Modfit LT was applied to the DNA histogram data. Modfit identifies the G1 and G2 peaks of DNA histograms acquired by flow cytometry and fits established cell cycle models to these peaks in addition to the S phase “peak”. The area under the curve is calculated via this model, thereby obtaining relative proportions of each cell cycle phase within the population.

### Immunofluorescence

Cells were fixed using 4% parafolmaldehyde for 15 minutes at room temperature and permeabilized using 0.25% Triton X-100 (TX) for 15 minutes. Blocking was performed in 10% donkey serum in 0.05% TX for 30 minutes followed by primary antibody incubation which was performed overnight at 4°C in blocking buffer with primary antbodies. Secondary antibody incubation was performed for one hour at room temperature in the dark with appropriate antibodies diluted in blocking buffer. Nuclear staining was performed by incubation with Hoescht stain in PBS for 5 minutes. Pictures were taken using Olympus IX81 inverted microscope and Metamorph imaging software.

### Statistical Analysis

Differentiation results are presented as averages of 6 separate independent experiments. Error bars represent SEM. Kriskal-Wallis test was used to determine statistical significant difference between the DE induction treatments. Additional Mann–Whitney U tests were used for post-hoc comparison with Bonferroni correction of the α.

### Mathematical Analysis

#### Principal Component Analysis (PCA)

The gene expression data containing the dynamics of the differentiation markers across the four stages of differentiation and the four conditions was analyzed using PCA. The data was preprocessed by mean centering and variance scaling across each transcription factor. PCA was done on this data in MATLAB R2010 by using the *princomp* option. It was found that the first two principal components (PCs) explained greater than 67% of the variance in the data for all the PC analyses performed. Therefore, two PCs were retained in the final analysis.

#### Clustering techniques

k-means clustering was used to identify transcription factors (TFs) that showed similar patterns of expression across the four stages independently for each condition. MATLAB function *kmeans* was used with correlation distance as a metric for clustering. The quality of the resulting clusters was judged by the Silhouette value (*S_i_*). A threshold of 0.6 was selected for *S*_i_, and the number of clusters *k* which gave all *S*_i_ values greater than 0.6 were determined. Hierarchical clustering was done on the entire dataset (all conditions together) to further classify the dynamics. MATLAB functions *pdist* and *linkage* were used to perform the analysis on the mean expression data and the results were represented as a clustergram. The tree generated using other linkage measures were found to be similar with a cophenetic correlation coefficient greater than 0.9.

#### Partial Least Squares Regression (PLSR)

PLSR was performed to find which of the earlier markers showed the highest correlation with *INS* upregulation. The gene expression data was gathered in matrix, *X*. *INS* was chosen as the output, *Y*, and the remaining transcription factors acted as the predictors. MATLAB function *plsregress* was used. The data was mean centered and variance scaled. The PLSR analysis results in a *BETA* vector of coefficients which describes the following relation between *Y* and *X*: 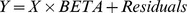



Here, *BE*T*A* is a (*n*+1) dimensional vector with the first entry as the intercept and the remaining *n* entries as the coefficients denoting the linear dependence of *I*N*S* on each TF.

## Results

### Pancreatic Differentiation of hESCs

**Figure 1:**
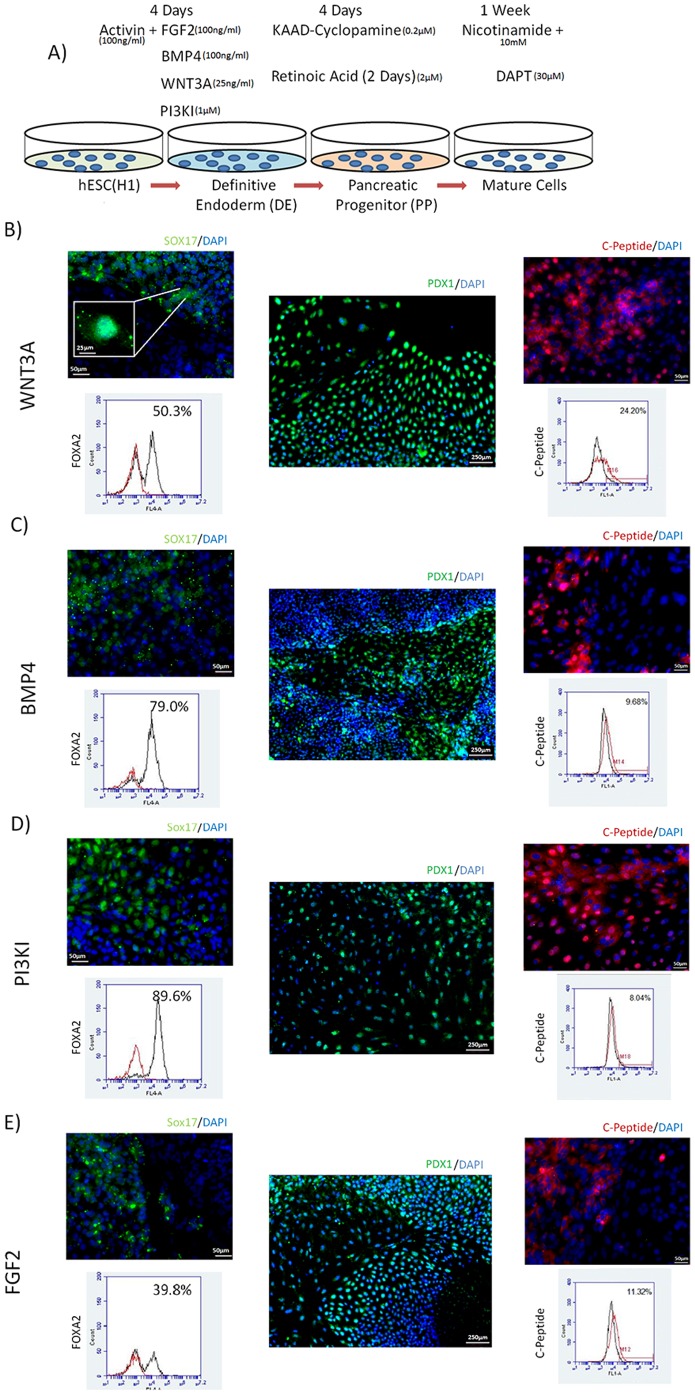
Multi-stage Differentiation System. **(A)** Schematic representation of multi-stage differentiation system. Detailed media formulation found in Supp table 1. DE was induced by modulation of nodal pathway simultaneously with one of four alternate pathways. PP was achieved by SHH inhibition along with retinol signaling. Maturation was induced by notch inhibition. Differentiation using WNT3A **(B)**, BMP4 **(C)**, PI3KI **(D)** or FGF2 (E) at DE stage. IF pictures show nuclear staining of SOX17 (green) and Flow cytometry shows yield of FOXA2 after DE induction, followed by nuclear PDX1 IF pictures (purple) after PP induction and cytoplasmic C-Peptide IF (red) expression yield as measured by flow cytometry after maturation.

A multi-stage directed differentiation protocol was used to induce the hESCs to pancreatic lineage (Fig. 1). The first step was to induce DE through multiple alternate pathways, which was achieved by exposure to Activin in combination with one of the four other growth factors and molecules that modulate alternate pathways for DE induction.

While Activin alone can induce DE, it is typically combined with different molecules to increase the efficiency of induction. In pancreatic differentiation studies, DE is most commonly achieved by combination of Activin A with WNT3A[13], BMP4[9], PI3K inhibitor[18] or FGF2[19].

After 4 days of DE induction Activin A and other inducers were removed and all groups were exposed to the same subsequent signals as follows (Fig. 1): for PP induction Cyclopamine was added alone for two days and in combination with retinoic acid for two additional days; cells were then exposed to nicotinamide alone for 2 days and nicotinamide and DAPT for up to one week for the maturation stage.

### Pancreatic maturation of hESCs is sensitive to the initial pathway of endoderm induction

**Figure 2:**
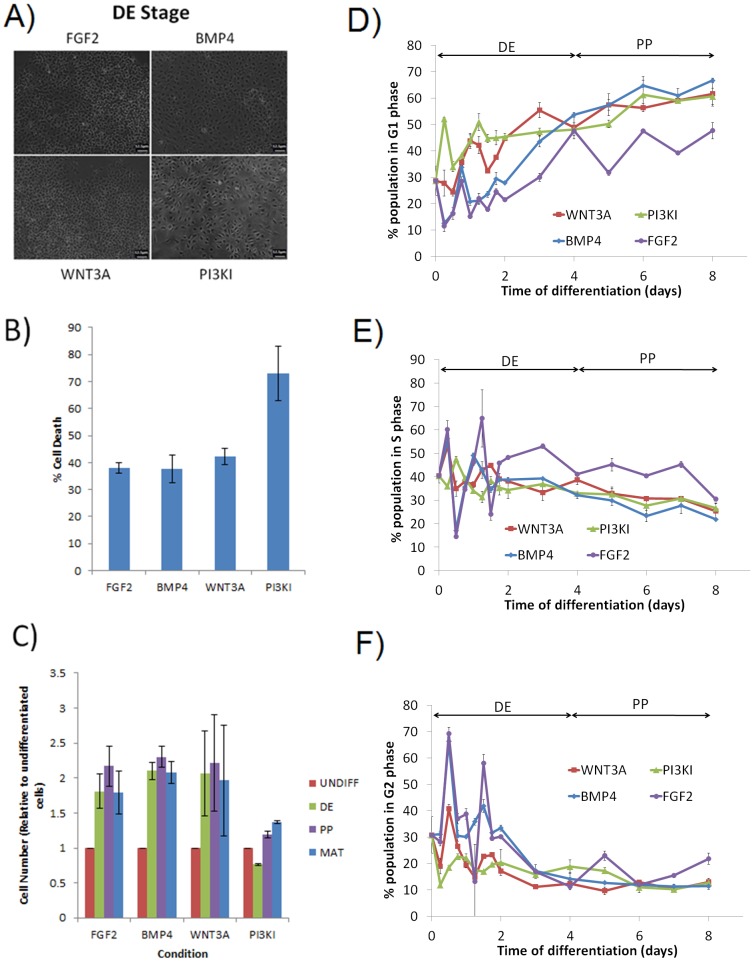
Cell Proliferation and Death Morphological analysis of the cells after **(A)** DE induction showing heterogeneous populations under all conditions (Scale bar: 12.5 µM), and **(B)** Cell death after 24 h of DE treatment. Death was comparable in all groups, except for PI3KI which resulted in considerably higher death **(C)** Increase in cell number was observed after DE and PP stage. Beyond the PP stage there is a slight decrease in cell number for all conditions except PI3KI. **(D–F)** Cell cycle analysis of the differentiating cellular population under different conditions, as analyzed and quantified by flow cytometry. Shown is the fraction of the population in the G1 **(D)**, S **(E)**, and G2/M **(F)** phases of the cell cycle. Data are represented as mean +/− STDEV.

Morphological examination of the matured cells exposed to alternate DE induction pathways revealed heterogeneous populations of cells in all conditions (Fig. 2A) containing groups of cobblestone like cells indicative of endoderm morphology; however, PI3KI cells appeared to be larger than other groups. To determine if this was attributed to cell confluence, and to analyze the system more thoroughly, we studied proliferation, apoptosis and dynamics of cell cycle under different induction conditions. Cell death at DE stage was comparable for all conditions except PI3K inhibition, which elicited high cell death (Fig. 2B). This is evident in Fig. 2C which shows a drop in cell number in PI3KI-DE. However, cell cycle dynamics (Fig. 2D–E) confirm a proliferative population, with similar dynamics between PI3KI and WNT3A conditions. Analysis of the cell cycle clearly indicates a maturing population of cells, transitioning from a dominant S phase to a dominant G1 phase, representative of mature cells [20]. As expected, undifferentiated hESCs (time = 0) have a short G1 phase as exhibited by a low sub-population (∼27%) in the phase, with subsequent increase in G1 residence time with differentiation (Fig. 2D). While the residence times of the S and G2/M phases are not expected to significantly change with differentiation, the fraction of the population in these phases decreases to compensate for the increased G1 phase (Fig. 2E, F). Compared to the PI3KI and WNT3A conditions, the kinetics of this transition, from dominant S to dominant G1, is very slow for the BMP4 and FGF2 conditions during initial DE induction, as exhibited by a small fraction of the population in G1 up until day 2 (Fig. 2D). The G1 population then increases until it reaches a level comparable to the WNT3A and PI3KI conditions at the end of DE induction (day 4). This is reflected in the proliferation data (Fig. 2C) showing an almost identical behavior between WNT3A, BMP4, and FGF2 at DE.

**Figure 3:**
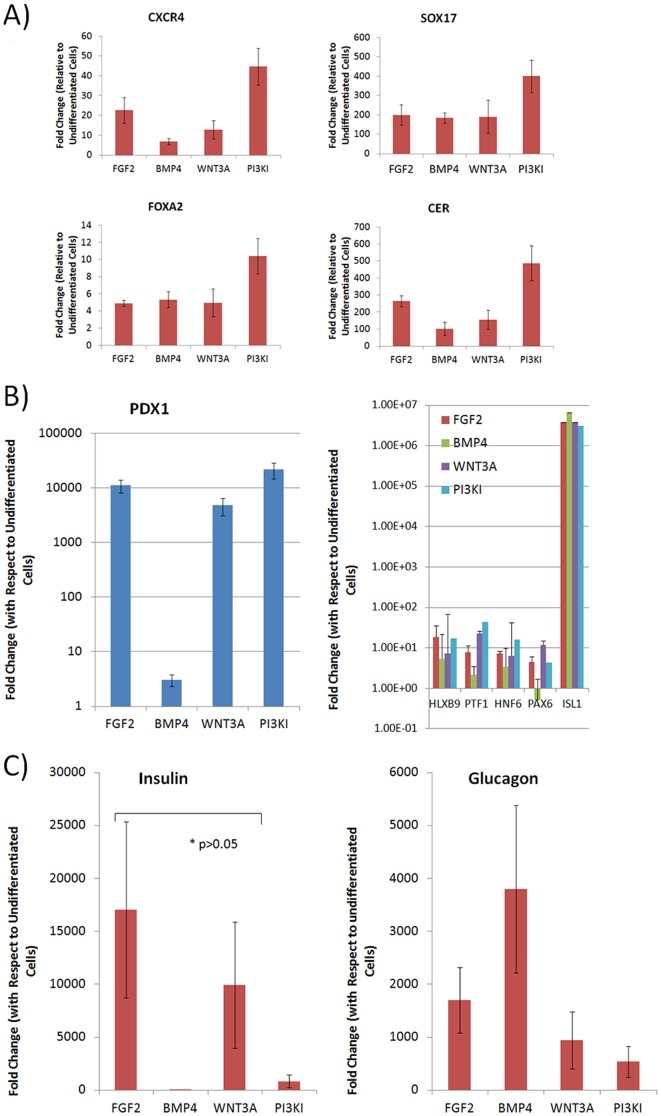
Stage Specific Marker Expression. Relative expression of **(A)** DE specific markers after DE induction under all differentiation conditions. Upregulation was obtained for all groups with PI3KI consistently yielding highest expression. **(B)** PP specific markers after PP induction for all DE derivatives with upregulation obtained for most markers under all conditions, except for BMP4 which consistently resulted in lowest upregulation. **(C)** Pancreatic hormone expression after maturation for all groups with WNT3A and FGF2 groups achieving highest upregulation of *INS* (p>0.05), while BMP4 obtained lowest *INS* upregulation but highest *GLUC* expression. Data are represented as mean +/− SEM.

In order to confirm differentiation after DE induction, immunofluorescence (IF) and flow cytometry for SOX17 and FOXA2 was performed for all groups after 4 days of treatment. Both transcription factors were found to be expressed in all groups (Fig. 1A–D) with yield of FOXA2 positive cells ranging from 40–90%. qPCR was performed to examine expression of stage specific markers *CXCR4*, *SOX17*, F*OXA2* and *CER*. As illustrated in Fig. 3A, upregulation of these markers was obtained under all differentiation conditions, with PI3KI consistently eliciting the highest upregulation, achieving close to 50 fold increase in *CXCR4*, 400 fold increase in *SOX17*, 10 fold increase in F*OXA2* and 500 fold increase in *CER*.

Upon pancreatic induction, all the induction conditions show expression of PP marker PDX1 by IF (Fig. 1A–D). This was further confirmed by qPCR for *PDX1*, which showed that with the exception of BMP4, all other conditions strongly expressed *PDX1* (Fig. 3B). A notable increase of other PP markers was also observed, particularly *ISL1*. BMP4 treated cells, however, consistently showed either comparable or lower upregulation of PP markers than the other groups. BMP4 treated cells additionally showed downregulation of *PAX6*.

At the last stage of differentiation, IF and flow cytometry confirmed expression of C-peptide for all groups with yields ranging from 9–24% (Fig. 1A–D). Detailed gene expression for mature β cell markers (Fig. 3C) revealed the highest *INS* mRNA upregulation under WNT3A and FGF2 conditions, both of them achieving over 10,000 fold increase compared to undifferentiated cells, with no statistical difference between them. While BMP4 condition showed the lowest (11 fold) upregulation of *INS*, it was the highest in upregulation of *GLUC* mRNA (Fig. 3C). It is noteworthy that there was considerable variability in the results from the different experiments, as observed by the error bars, attributed to population heterogeneity and variable response to global inductive cues [21], [22]. However, despite the variability, all experiments consistently showed a similar trend where highest insulin upregulation for every experiment was obtained in FGF2 of WNT3A treated cells, while BMP4 consistently lead to insignificant insulin upregulation. Results from individual experiments can be found in figure S1.

The above analysis clearly indicates that the initial pathway of endoderm induction plays a crucial role in subsequent maturation of the cells towards pancreatic lineage.

### Alignment of in vitro differentiation with in vivo organogenesis

Research over the last decade has established the advantage of directed differentiation of hESCs following the sequence of *in vivo* development. Hence, there is an increased emphasis on aligning the *in vitro* differentiation dynamics to *in vivo* organogenesis events. Accordingly, we analyzed the alternate pathways of endoderm induction and subsequent maturation in the light of differentiation dynamics.

**Figure 4:**
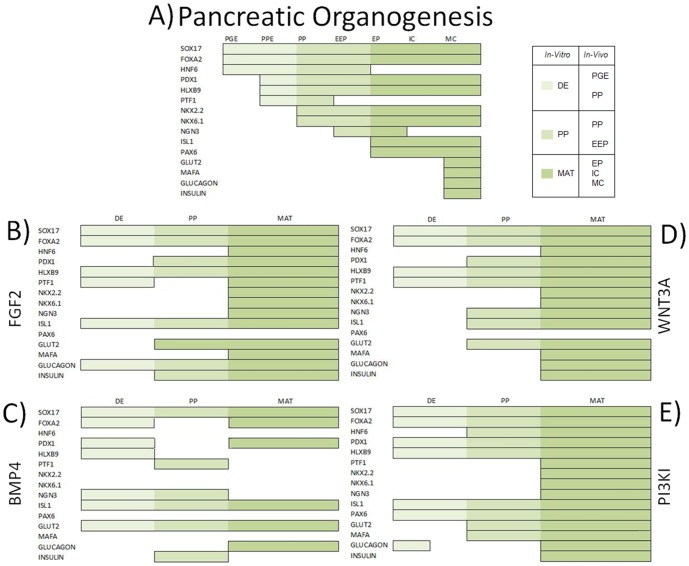
Marker Progression. A representative sample (based on *INS* expression) for each group was analyzed and compared to in-vivo **(A)** pancreatic development [24] in order to identify which DE pathway modulation(s) lead to better resemblance to pancreatic organogenesis. Similarities can be observed when DE induction is achieved by modulation of **(B)** FGF2, **(C)** BMP4, **(D)** WNT3A and **(E)** PI3KI while we observed that marker progression greatly differs under BMP4 induction. The different stages of pancreatic development were grouped to represent the 3 stages of the differentiation protocol. Primitive gut endoderm (PGE) and prospective pancreatic endoderm (PPE) represent definitive endoderm induction (light green) pancreatic progenitor (PP) and early endocrine progenitors (EEP) represent pancreatic progenitor induction (medium green) and endocrine progenitors (EP), immature β- cells, mature β- cells (MC) represent the maturation stage (dark green).

Pancreatic development can be broadly divided into 7 stages, each characterized by specific transcription factors. The first stage is primitive gut endoderm (PGE), from which pancreas, lung, thyroid, thymus, parathyroid, and liver are derived[23], followed by prospective pancreatic endoderm (PPE) containing prospective ductal, endocrine and exocrine pancreatic cells. The next step is the pancreatic progenitor (PP) stage marked by the transient expression of PTF1 and is followed by appearance of NGN3 expressing early endocrine progenitors (EEP). EEP develop into endocrine progenitors (EP), from which all the islet cell types develop, including α, β, γ, δ and ε cells. From here disappearance of NGN3 expression marks emergence of immature β- cells which mature into functional, *INS* expressing β-cells[24]. To draw a parallel to our 3-stage differentiation protocol, we combined specific developmental stages as follows: DE stage includes endoderm, PGE, and PPE; PP stage includes PP and EEP induction; and the maturation stage includes EP induction, immature β- cells and β- cell maturation. Fig. 4A illustrates a qualitative measure of the expression patterns of stage specific transcription factors across different stages of development as gathered from literature [23], [24]. Fig. 4 (B–E) presents parallel transcription factor dynamics for hESC differentiation under different DE induction conditions as observed in a representative sample with *INS* expression closest to the mean. For the purpose of this study, we defined presence of a marker as 10 fold or higher upregulation as observed by qPCR in order to account for experimental error. Overall the FGF2, WNT and PI3KI conditions were found to exhibit similar trends as *in vivo* development, only with some minor differences. For example, *PTF1* is known to be an early and transient marker of pancreatic commitment, preceding *PDX1* expression. While both FGF2 and WNT conditions show a gradual increase in *PTF1*, under PI3KI conditions *PTF1* comes up very late even though *PDX1* expression is detected much earlier, even at the DE stage. On the other hand *PAX6*, which is expressed early in α cells and later in the entire islet [25], and has been suggested to be a key component of glucagon secretion [26], is prominent in PI3KI conditions from an early stage (DE) and increases with maturation. BMP4 condition, however, was found to be an outlier, as it did not align with either *in vivo* sequences or any of the other conditions.

### BMP4 induced DE cells exhibit a divergent maturation dynamics

#### Analysis by hierarchical clustering

**Figure 5:**
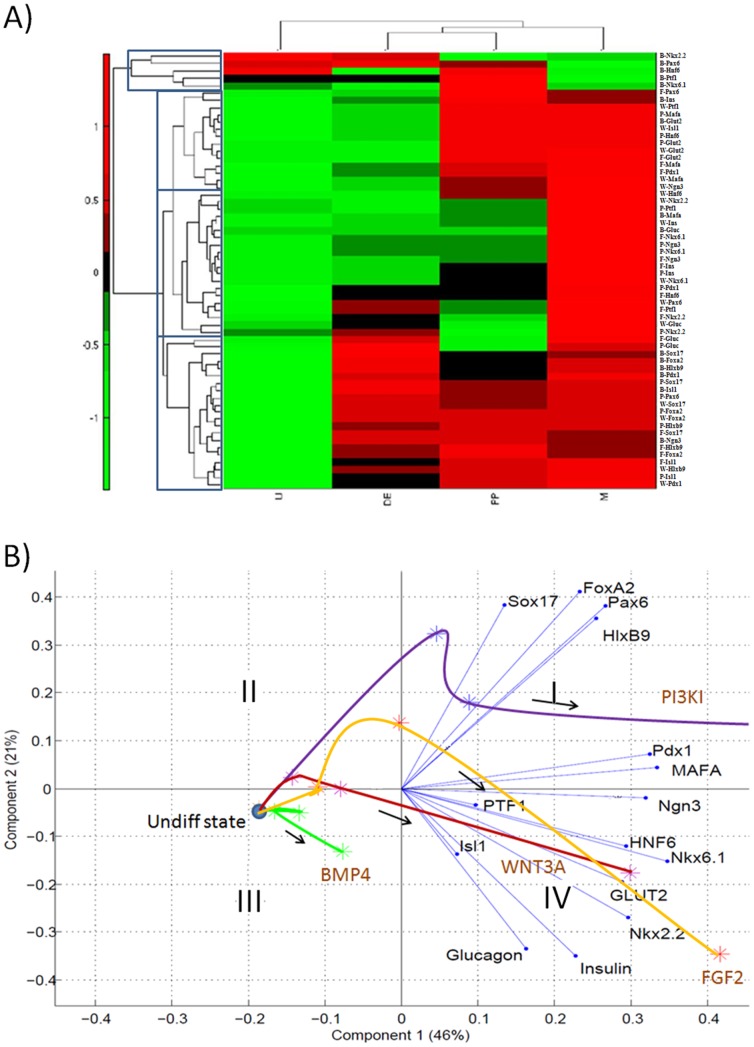
Transcription factor dynamics. **(A)** Heat map for the entire data set of genes and conditions illustrating marker progression throughout differentiation stages. The genes are organized according to the expression clusters found through hierarchical clustering. The treatments are denoted on the right hand side as prefixes to the gene names. BMP4 induction condition typically was found to cluster separately from the rest. Hierarchical clustering was performed on the mean centered and variance scaled data of transcription factor dynamics across all the four DE induction conditions. **(B)** Biplot of transcription factor dynamics assessed by principal component analysis on the mean data-set. The first component shows a demarcation of the undifferentiated and differentiated states. The second component divides the markers according to their expected appearance during *in vivo* differentiation. The PI3KI curve moves closer to the DE markers, BMP4 curve does not perform well and the WNT3A and FGF2 curves show successful pancreatic maturation.

In order to resolve the differentiation dynamics further, we performed hierarchical clustering of 15 stage specific transcription factors measured over 4 time points under the 4 DE induction conditions. Fig. 5A shows a heat map of transcription factor dynamics. Hierarchical clustering of the transcription factor dynamics identified four clusters of TFs, of which the most striking was the one formed under BMP4 induction (*NKX2.2*, *PAX6*, *HNF6*, *PTF1*, *NKX6.1*). These factors were rapidly down-regulated with differentiation induction, the highest expression being in the undifferentiated cells. It is important to note that the data in Fig. 5A is presented as relative expression; hence, even though the absolute gene expression for undifferentiated cells were the same under all conditions, the differences in the heat map arises from the normalization. Additional graphs illustrating expression patterns of each individual marker are shown in figure S2. The aforementioned cluster branched separately from all of the remaining clusters indicating the difference in transcriptional activation following BMP4 treatment. Overall, many of the PP markers were higher at the DE stage under BMP4 treatment while the later markers were not upregulated upon maturation. On the other hand, FGF2, WNT3A, PI3KI treatments followed the pancreatic organogenesis closely as shown by clusters 2 to 4. On closer inspection, it was found that 67% of the markers assayed for are regulated in a similar manner under FGF2 and WNT3A pathway modulation, representing the largest similarity between the pathways studied. PI3KI leads to 47% and 40% similarity with WNT3A and FGF2 respectively, and the 3 pathways regulate 33% of the genes in a similar way. BMP4 treatment results in the most dissimilar gene patterning, sharing only 7% similarity with FGF2 and 20% with both WNTA and PI3KI. Additionally, the magnitude of upregulation of genes assayed for, including *INS*, was comparable for the FGF2 and WNT3A conditions at all stages of differentiation. Taken together, these results suggest similarity in pancreatic maturation stages when FGF and WNT pathways were modulated for initial endoderm differentiation.

In the hierarchical clustering formulation, each of the markers was treated separately under each induction condition giving rise to a total of 60 marker-condition pairs. However, in order to compare the dynamics of differentiation, it will be advantageous to look at the dynamics in the same space of transcription factors. Hence, we projected each of the induction condition in the same space of the transcription factors using principal component analysis (PCA). However, PCA extracts new orthogonal directions from the original space which are combinations of these markers.

#### Examination by principal component analysis

PCA allows visualization of multidimensional data in a new orthogonal coordinate space of PCs, and often the first few PCs explain most of the variation in the data. In our case, we found that the first two components explained 67% of the variation in the data, which is significant for biological systems as the remaining variability can often be attributed to noise. Fig. 5B shows a biplot where the time points for each of the four induction conditions are plotted in the PC space with the original variables (TFs) overlayed onto the plot. The first PC divides the region into the undifferentiated state (III) and differentiated state (I & IV). The second PC further splits it into early markers (I & II) and the late markers (IV). Ideally, to mimic pancreatic development, the cells must proceed from the III quadrant (undifferentiated state) to IV (mature hormone expressing cells) via I (DE stage). Except BMP4, all the other induction methods closely follow this path. It is found that WNT3A and FGF2 follow similar paths ending up closer to the *INS* and *GLUC* axes while PI3KI deviates significantly. PI3KI treatment still favors the DE markers like *SOX17* and *FOXA2* and some late markers like *PAX6*, *HLXB9* and *PDX1* during the final stages. However, PI3KI derivatives fail to perform well with respect to the important mature markers like *INS* and *GLUC*. BMP4 derivatives perform very poorly with respect to *INS* expression, which is accompanied by low expression of essential β-cell regulatory factors such as PAX4 [27], [28] (figure S3).

Diverse analysis of the experimental data leads to a similar conclusion: BMP4 induction is less suitable for pancreatic β-cell maturation. This is primarily because of low *INS* expression in the mature phenotype along with lack of timely upregulation of intermediate transcription factors known to be associated with β-cell development. However, BMP4 treatment resulted in high *GLUC* expression, although other associated α-cell markers were not synergistically upregulated.

### K-means clustering of individual pathways reveal WNT3a to be more consistent with development

Our next goal was to determine which of the remaining conditions are more suitable to drive pancreatic maturation. One way to assess this is to find representative TFs that show coherent expression dynamics. To address this question we scrutinized each of the pathways individually through K-means clustering of each induction condition.

**Figure 6:**
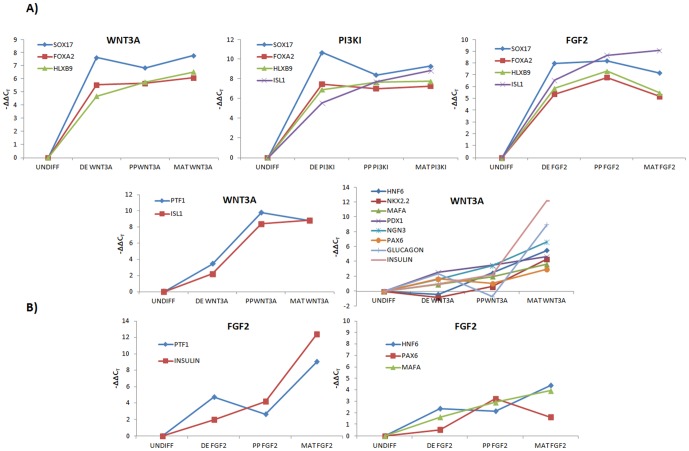
Significant K-means clusters. Clusters obtained for each induction condition. **(A)** WNT3A **(B)** PI3KI **(C)** FGF2 and **(D)** BMP4. The k-means clusters show close similarity of our induction conditions WNT3A and FGF2 with pancreatic organogenesis and PI3KI with definitive endoderm commitment. The markers *SOX17, FOXA2, HLXB9* are closely regulated under all the induction conditions.

As shown in Fig. 6A, *SOX17, FOXA2, HLXB9* were co-regulated under WNT3A, FGF2 and PI3KI conditions. These markers indicate the DE and dorsal pancreatic endoderm. This combination of *SOX17*, *FOXA2* and *HLXB9* was repeated in all the above induction conditions, indicating that each of these treatments is efficient for activating the primary DE transcriptional machinery and that at the later stage transcriptional activation is different. These markers were consistently expressed through all the differentiation stages. In addition, PI3KI and FGF2 clusters also contained *ISL1*. However, no other coherent cluster was obtained for the PI3KI condition, indicating lower alignment with developmental dynamics towards the later stages of maturation.

Additional clusters containing many later markers were obtained for WNT3A and FGF2 as shown in Fig. 6B. One among these was *PTF1* and *ISL1* under WNT3A which arise in the pancreatic precursor cells during the early bud-stage. Other late markers such as *PAX6*, *PDX1*, *MAFA*, *GLUC*, *INS*, *NGN3*, *HNF6*, and *NKX2.2*, which are expressed in the NGN3+ cells maturing to the β-cell stage [29], were also identified under WNT3A treatment. These later markers show continuous rise in expression across the stages. Therefore, it reinforces the observation that early WNT3A induced cells were found to closely shadow the *in vivo* embryonic transcriptional dynamics. For FGF2, clusters containing small number of late markers were identified as shown in Fig. 6B. Two groups were identified, one containing *PTF1* and *INS* and the other containing *HNF6*, *PAX6* and *MAFA*. However, FGF2 contained far less coherent markers at the later stages than WNT3A.

The above analysis indicates that modulation of Activin with WNT and FGF2 are likely routes to pancreatic β-cells, although WNT pathway is identified to be the most suitable because of the co-regulation of important markers during each stage of the differentiation process. Furthermore, this comparison reveals that even though the expression of DE and PP markers are quite similar for all the induction conditions at the end of DE stage, they deviate significantly upon maturation. This is suggestive of cellular ‘memory’ of pathway of initial induction even after phenotypic maturation.

### PP markers, and not DE markers, are reliable predictors of islet maturation

The above results establish that different pathways of endoderm induction of hESCs have a significant influence on the cells' subsequent mature phenotype and functionality. Another way of looking at it is that efficiency of endoderm commitment, as analyzed by current markers, is not indicative of an efficient pancreatic maturation. The next question thus is whether any of the early or intermediate stages can reveal the potential for cellular maturation to islet cell types.

**Figure 7:**
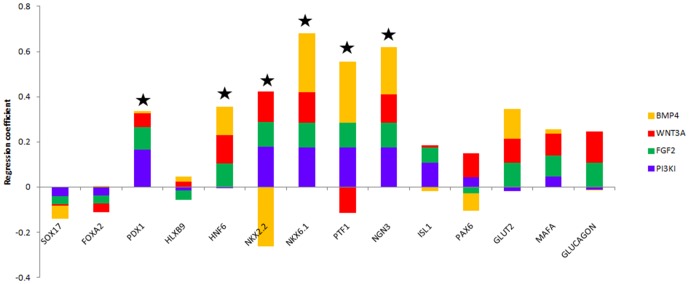
Predictors of *INS* expression. Partial least squares regression performed on the mean expression. Most PP markers show high degree of correlation to *INS* expression while there is no significant dependence on the DE markers. WNT3A and FGF2 conditions gave positive coefficients with most of the PP and mature markers indicating that these conditions are optimal for *INS* expression. R^2^ values were above 0.995.

We addressed this by performing partial least squares regression (PLSR) analysis on the mean TF expression data to identify which early TFs, if any, were predictors of *INS* expression. Here we are seeking the TFs that showed the most significant correlation to INS expression over all the time points of the differentiation trajectory. The correlation of each of the TFs with *INS* for each induction condition is represented in Fig. 7 as associated regression coefficients. It is found that most of the PP markers show high degree of correlation to *INS* expression while there is no significant dependence on the DE markers analyzed. None of the early DE markers analyzed show a positive correlation to *INS* across all the induction conditions. The intermediate PP stage markers, including *PTF1, PDX1*, *HNF6*, *NKX2.2*, *NKX6.1*, and *NGN3*, are better predictors of *INS*. Also, WNT3A and FGF2 conditions gave positive coefficients with most of the PP and mature markers indicating that these conditions are optimal for *INS* expression. It is also observed that under BMP4 and PI3KI, the markers *NKX6.1*, *PTF1* and *NGN3* gave strong positive correlations indicating that these markers are in fact strongly associated with *INS* even under low *INS* upregulation. In addition, we analyzed the expression of PP markers after DE induction and found high expression of *HLXB9*, *PTF1* and *ISL1* at this early stage under some of the conditions for the selected sample. Interestingly, *PTF1* resulted in high upregulation under FGF2 and WNT3A, which resulted in highest *INS* upregulation. This observation, combined with the fact that *PTF1* expression is highly correlated to *INS* expression under many conditions from PLSR, suggests that analysis of *PTF1* expression after DE induction could be used as a determinant of pancreatic potential.

## Discussion

This study analyzes and compares the potential of pancreatic maturation of DE derivatives obtained from hESCs following alternate pathways. Our primary goal was to determine if potential for pancreatic maturation was sensitive to the pathway of initial DE commitment, and if so, to determine which pathway is most supportive of pancreatic maturation. In order to do so, we have chosen the most commonly reported hESC cell line for pancreatic differentiation (H1) for our analysis.

We chose to analyze those DE induction pathways which have been most commonly reported in literature for pancreatic differentiation of pluripotent stem cells. There have been reports of successful DE induction following alternate routes which have not been considered in the current study. For example, identification of small molecules has shown great promise as a cost effective alternative to expensive growth factors. While these molecules have not been directly compared in our protocol, many of these molecules modulate similar pathways as discussed here. Some examples include 1m and CHIR99021 which act by inhibiting GSK3β through WNT3A [30], [31]; and IDE1 and IDE2 which modulate the nodal pathway through activation of the TGF-β signaling pathway, similar to Activin [32]. In addition, we have recently reported the sensitivity of endoderm differentiation to substrate physical properties when cultured on fibrin [33] and alginate gels [34]. However, the exact mechanism involved in such induction of differentiation through insoluble cues has not yet been elucidated.

Importantly, we found that the yield of mature *INS* expressing cells was sensitive to the pathways for initial DE induction. Our analysis suggests that BMP4 signaling is not conducive for pancreatic β-cell differentiation of hESCs. Even though other studies have used BMP4 to achieve DE differentiation with subsequent maturation to pancreatic lineage [8], [9], [35], in our studies BMP4 derived DE derivatives were found to exhibit a stronger potential for *GLUC* expression when subjected to our maturation protocol. Several reasons could be attributed to this difference in the results. It is important to highlight that while these studies also use BMP4 at early stages of differentiation, there are obvious differences in the remaining differentiation protocol. Phillips et al [8], [35] reported the use of BMP4 in combination with Activin in early stages of differentiation; however, in later stages they use FGF, IGF, HGF, and VEGF, amongst other factors. Their differentiation protocol is based on pancreatic differentiation from adult pancreatic ductal cells, while our protocol is based on recapitulation of events present during *in vivo* pancreatic organogenesis.

The earliest effect of BMP pathway modulation during pancreatic development occurs at early DE development, where in combination with Activin and FGF2, BMP4 signaling specifies DE induction [8]. Also, at the earliest stages of differentiation, BMP4 accelerates the downregulation of pluripotency genes and upregulation of mesendodermal genes like *BRACH* [10]. However, later effects of BMP4 are inhibitory of pancreatic differentiation and strong inducers of hepatic differentiation [7]. In our experiments we see BMP4 to consistently induce lowest upregulation of DE, PP and mature β-cell markers which could indicate residual BMP4 signaling from DE induction even after removal of BMP4 from media. This is consistent with several pancreatic differentiation studies that use BMP4 at DE induction stage, but use noggin, a BMP pathway inhibitor, at later stages of differentiation [35], [36]. From marker progression analysis (Fig. 4) we see that in BMP4 treated cells, *NGN3* peaks early during DE induction, with maintenance throughout the PP stage, and decreases during the maturation stage. A recent study has implicated temporal regulation of *NGN3* as an important determinant of cell type specification, with early expression favoring α-cell induction [37]. In agreement with this, we also see BMP4 cells do express highest levels of *GLUC* while exhibiting a very low upregulation of *INS* and other essential β-cell markers, including PAX4, NKX2.2 and NKX6.1[27], [28], [38]. These results suggest that DE specification signaling may prime cells for a particular mature endoderm cell type.

In a parallel work we tested possible combinations of growth factors for inducing DE and found the combination of Activin, FGF2 and BMP4 to give a high upregulation of *SOX17* and *CXCR4* compared to using FGF2 or BMP4 alone [17]. Recent work by Yu *et al*. [39] gives an explanation for this effect, with FGF2 sustaining Nanog expression and helping the BMP4 induced differentiation to shift towards endoderm as opposed to extra embryonic lineages. In a previous study by Vallier *et al.*, using Activin in combination with FGF2 and BMP4 resulted in similar differentiation into mesendoderm [40]. Based on this, we decided to mature the endoderm cells derived under combination of FGF2 and BMP4 towards pancreatic maturation. However, the level of *INS* upregulation upon maturation remained comparable to that of BMP4 treatment alone (data not shown). Thus, FGF2+BMP4 under high Activin seems to work well for DE induction, but may not be optimal for further maturation to the pancreatic lineages.

Our analysis further indicated modulation of WNT pathway to be most supportive of pancreatic maturation. Several studies establish the WNT canonical pathway as a potent endoderm inducer and its presence has been shown to stimulate expression of endoderm markers [24], [41], [42] while inhibition of the WNT pathways induces increase of cardiac markers [43]. WNT is therefore added during *in vitro* differentiation of hESCs particularly during the initial stages of mesendoderm induction. The canonical WNT pathway is found to cooperate with the Activin (SMAD) signaling pathways for the expression of mesendoderm specific genes [44]. However, WNT signaling must be suppressed at the later stages during differentiation to the posterior foregut endoderm [45], [46]. Our results show WNT3A DE derivatives to result in high *INS* expression levels and highest yield of C-peptide positive cells. In agreement with this, in a previous study, Nostro *et al*. found that at low concentrations, increasing canonical WNT pathway activation at the endocrine development stage gave higher upregulation of *INS* [35].

In addition, WNT3A and FGF2 shared most similarities in terms of gene expression patterns and magnitudes, suggesting similar transcriptional regulation. Also, the results of PCA showed that the trajectory of differentiation was very similar for these two conditions. Interestingly, both these conditions also lead to highest expression of *INS* mRNA levels with no statistical difference between them. While gene expression patterns were similar between WNT3A and FGF2, cell cycle analysis reveals substantial differences (Fig. 2). The length of the G1 phase for WNT3A treated cells increased at a faster rate than for FGF2, as shown by the higher proportion of the population in the phase. This longer G1 phase time is indicative of a more mature phenotype [20], [47]. Therefore, while both conditions give desirable mature gene expression, WNT3A is the preferred route for maturation based not only on gene expression but cell cycle behavior. The conclusion that WNT3A leads to better pancreatic differentiation potential is in agreement with a number of pancreatic differentiation studies that use WNT3A in combination with Activin A at the definitive endoderm stage, which have reported better yield, insulin expression and functionality after *in vivo* maturation than other pancreatic differentiation studies [3], [13], [14]. However, it is difficult to identify the source of this variability since most of these protocols are significantly different in terms of the growth factors and reagents used along with reported cell lines. Our studies, in a controlled platform, establish the significant effect of DE induction pathway alone on the potential for cell maturation.

Finally, our results highlight the insufficiency in analyzing DE markers at the DE stage as an adequate representation of cellular maturation potential. In our experiments, PI3KI consistently showed highest upregulation and yield of DE markers at the end of the DE induction stage. However, upon maturation, its potential for *INS* upregulation was lower than that of WNT3A and FGF2. Our correlation analysis also supported this observation, where we found the PP markers to correlate strongly with *INS*, but not the earlier DE markers. This indicates that analysis of PP markers is likely to give us information on the maturation potential of the differentiating cells, but analysis of the DE markers alone is unlikely to reveal such information. In addition, we suggest *PTF1* expression analysis after DE induction to be a potential candidate to determine pancreatic potential. *PTF1* expression showed a high degree of correlation to *INS* expression in our PLSR analysis. Consequently, *PTF1* expression appeared early in conditions resembling pancreatic progression, where *PTF1* expression appears at the prospective pancreatic endoderm stage.

## Conclusion

Multiple reports currently exist in literature on alternate pathways for endoderm differentiation, yet the effect of the pathway of endoderm commitment on late stage maturation, if any, remains unexplored. In this study we conducted a systematic investigation on the sensitivity of pancreatic maturation of hESCs to the pathway of endoderm commitment. Late stage differentiation was judged by gene expression levels of representative β-cell markers, since the field is as yet limited in demonstrating functionality of the hESC derived islet-like cells. This report highlights the importance of the pathway of endoderm differentiation - over efficiency of the differentiation - in shaping the potential for further differentiation and maturation.

## Supporting Information

Figure S1 **Insulin expression for individual trials.**
(TIF)

Figure S2 **Expression patterns of individual genes under all experimental conditions.**
(TIF)

Figure S3 ***PAX4*** **expression under all experimental conditions.**
(TIF)

Methods S1 **Detailed mathematical analysis methods.**
(DOCX)

Table S1 **Media formulations for all stages of differentiation.**
(DOCX)

Table S2 **Primers for quantitative RT-PCR.**
(DOCX)

Table S3 **Antibodies for flow cytometry and immunocytochemistry.**
(DOCX)
