# Supplementary figures and images for: Potential for Pancreatic Maturation of Differentiating Human Embryonic Stem Cells Is Sensitive to the Specific Pathway of Definitive Endoderm Commitment
Source: PLoS One. 2014 Apr 17;9(4):e94307. doi: 10.1371/journal.pone.0094307 (PMC3990550; doi:10.1371/journal.pone.0094307)

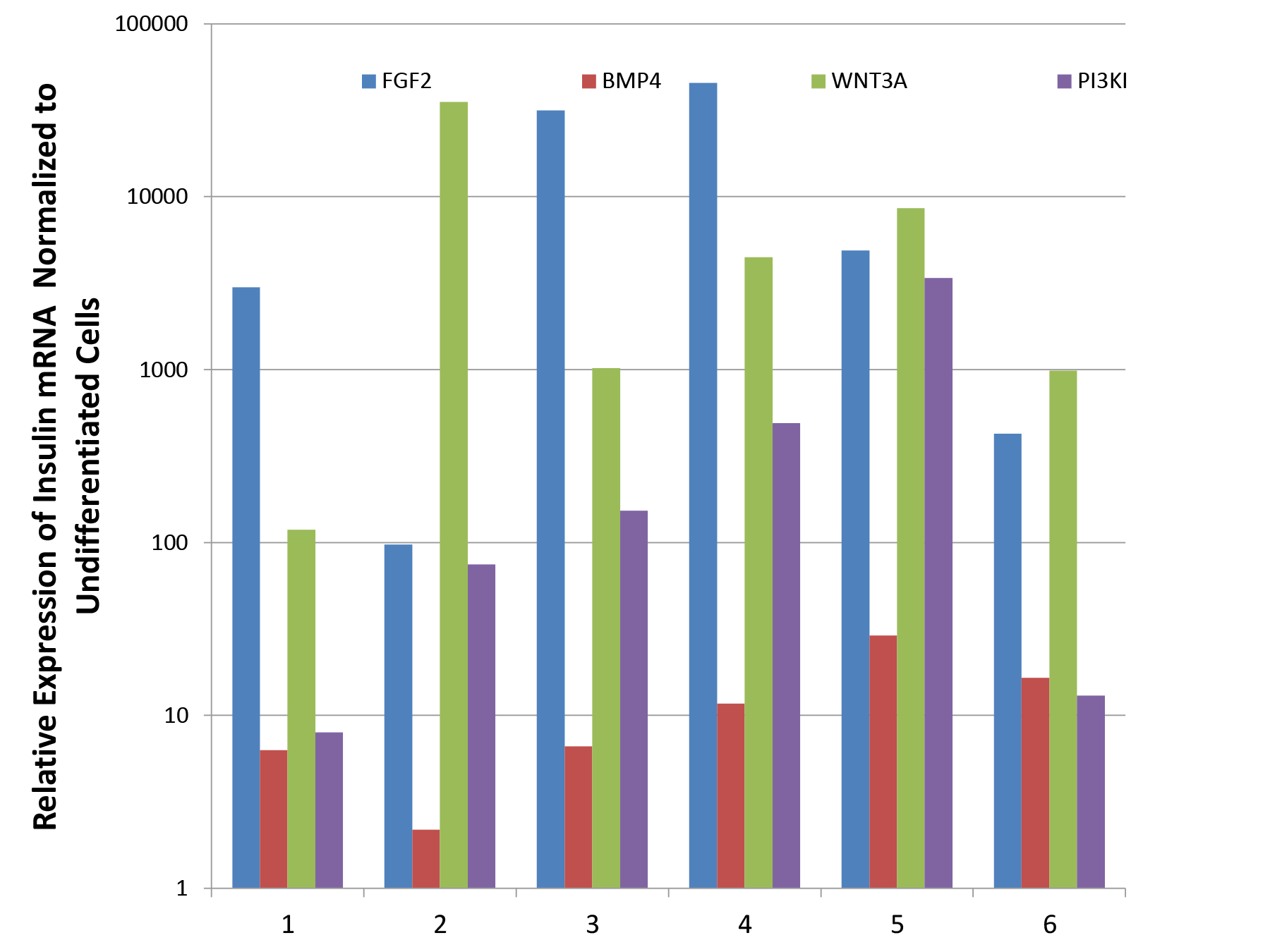

Supplement: Figure S1 — Insulin expression for individual trials. (TIF) [file pone.0094307.s001.tif]

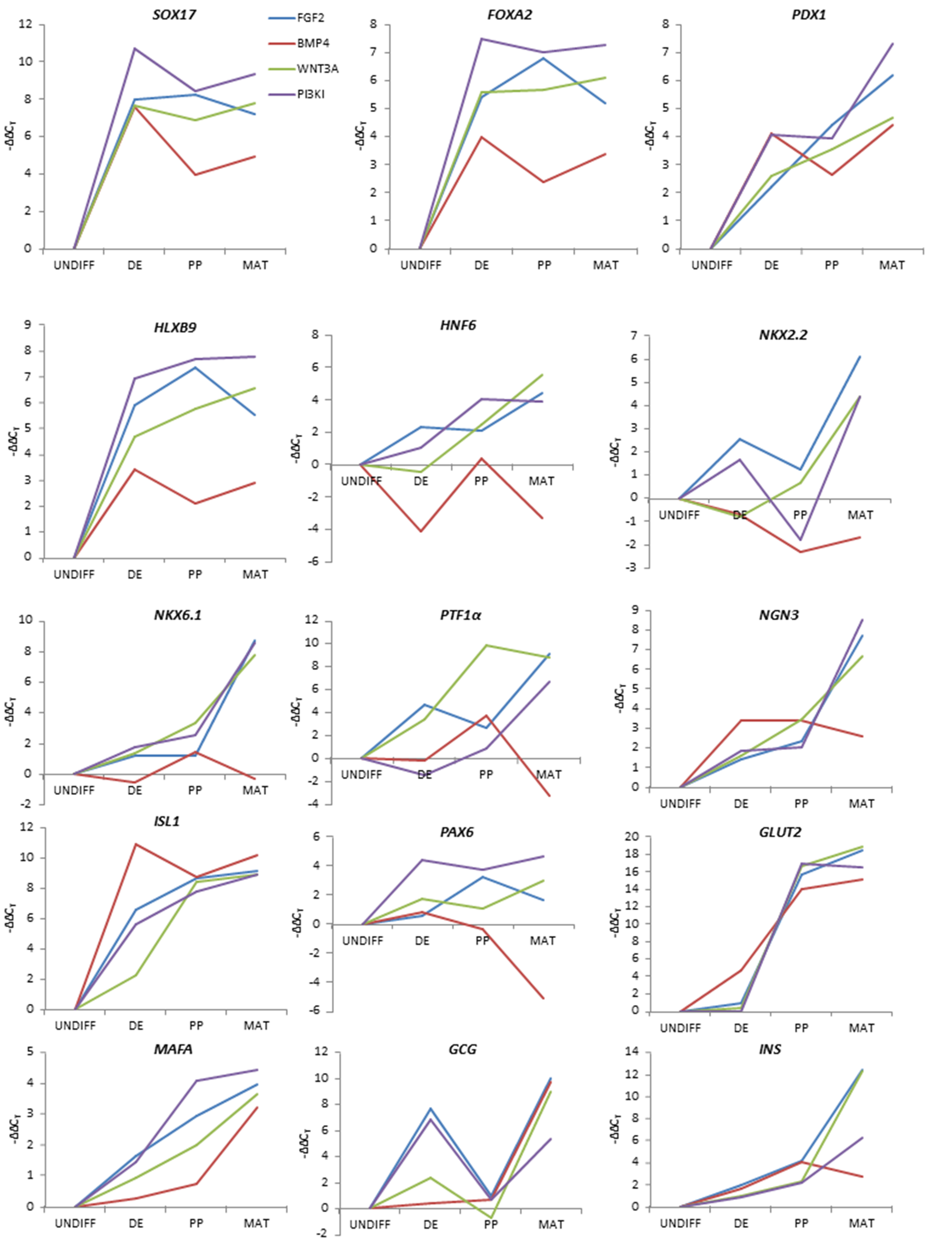

Supplement: Figure S2 — Expression patterns of individual genes under all experimental conditions. (TIF) [file pone.0094307.s002.tif]

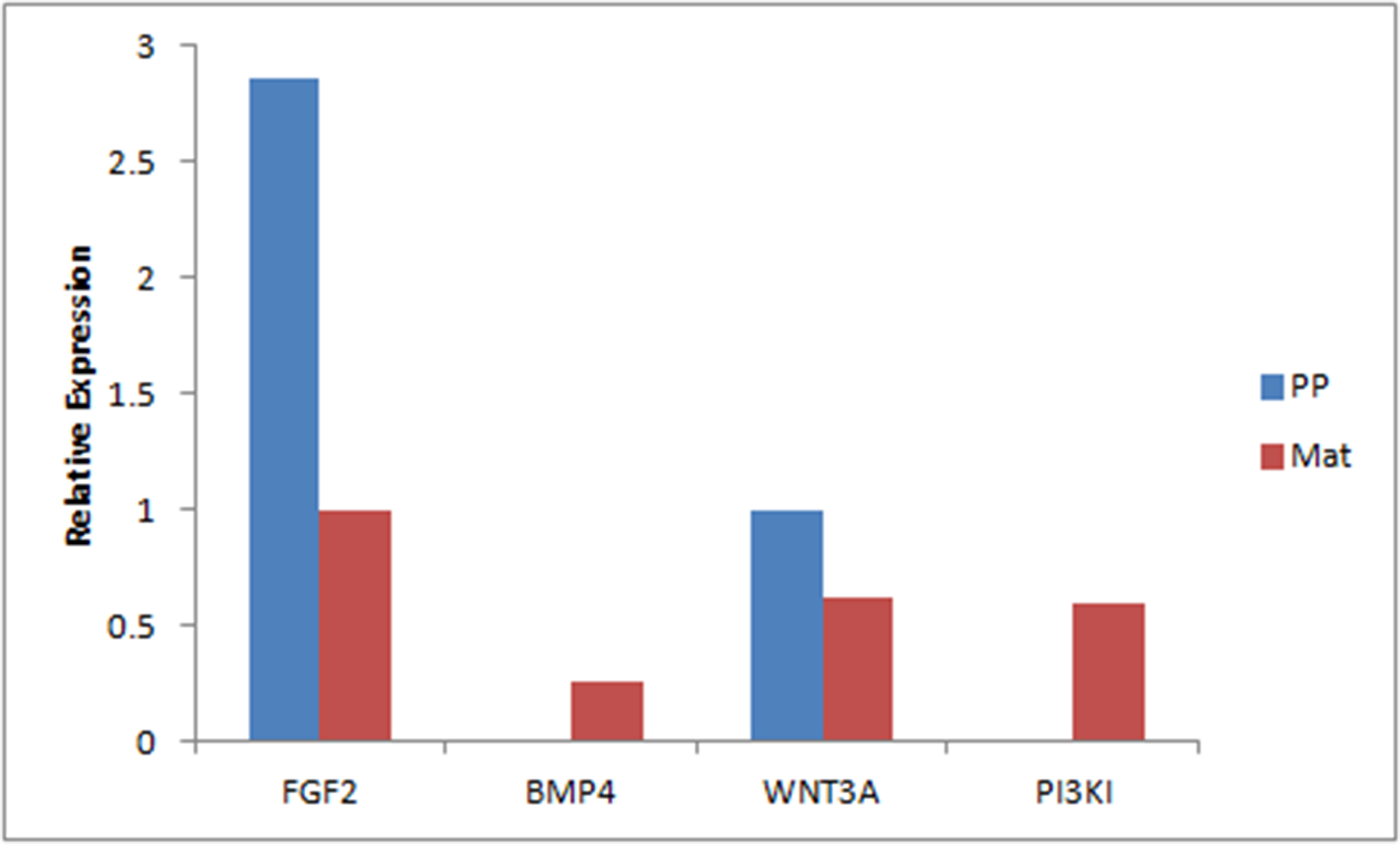

Supplement: Figure S3 — PAX4 expression under all experimental conditions. (TIF) [file pone.0094307.s003.tif]
