# Supplementary material for: Potential for Pancreatic Maturation of Differentiating Human Embryonic Stem Cells Is Sensitive to the Specific Pathway of Definitive Endoderm Commitment
Source: PLoS One. 2014 Apr 17;9(4):e94307. doi: 10.1371/journal.pone.0094307 (PMC3990550; doi:10.1371/journal.pone.0094307)
Supplement: Methods S1 — Detailed mathematical analysis methods. (DOCX) [file pone.0094307.s004.docx]

**Mathematical Analysis**

Principal Component Analysis (PCA)

The gene expression data containing the dynamics of the differentiation markers across the four stages of differentiation and the four conditions was analyzed using PCA. PCA transforms the data into an orthogonal co-ordinate system which helps to derive the relationships between the observations (time points) in the new orthogonal co-ordinate space of principal components. The derived principal components are functions of the original variables (i.e. TFs). For the purpose of our analysis, we generated expression matrix, *X,* with rows representing the transcription factors and columns as the four stages of differentiation. The matrix, *X,* was normalized by mean centering and variance scaling across each transcription factor. PCA was done on this matrix in MATLAB R2010 by using the *princomp* option. The method uses Singular Value Decomposition (SVD) to factorize the matrix into scores, F, and loadings, Q, matrices respectively (Abdi and Williams, 2010) such that $X^{T}=FQ^{T}$. The first PC explains the largest variation in the data and the next PC captures the next largest and so on. Each of these PCs are related to the $I$ eigen values of the correlation matrix, $XX^{T}$, arranged in the decreasing order. The percentage variance in the data captured by each principal component is thereby calculated using the formula,

$$\% var_{i}=\frac{\lambda_{i}}{\sum_{i=1}^{I} \lambda_{i}}$$

where, $\lambda_{i}$ is the $i^{th}$eigen value of the correlation matrix, $XX^{T}$. It was found that the first two PCs explained at least 67% of the variance in the data for all the PC plots in this paper. Therefore, 2 principal components were retained in the final analysis.

K-means clustering

k-means clustering was used to partition the data matrix of *n* TFs into *k* clusters such that each cluster represented TFs with coherent expression profiles across the four stages. Mathematically, each transcription factor is represented as a vector and L1-norm is used as the distance measure between them. MATLAB function *kmeans*, which is a two phase iterative algorithm, was used for the analysis. The algorithm minimizes the sum of vector-to-cluster centroid distances. The analysis was repeated 10 times with different random starting points so as to ensure that a global minimum is reached. The quality of the resulting clusters was judged by the Silhouette value. The Silhouette value for a member *i* in a cluster is defined as,

$S_{i}=\frac{(b_{i}-a_{i})}{\max(b_{i},a_{i})}$, where *a_i_* is the average distance from the *i*^th^ member to the other members in the same cluster as *i*, and b_i_ is the minimum average distance from the *i*^th^ member to members in a different cluster. We selected a threshold of 0.6 for *S*_i_, and determined the number of clusters *k* which gave all *S*_i_ values greater than 0.6. We also tested different distance measures and found similar clusters.

Partial Least Squares Regression (PLSR)

We performed PLSR to find which of the earlier markers showed the highest correlation with *INS* up-regulation. The matrix, *X,* was generated as described in PCA. Now, for PLSR, *INS* was chosen as the output, *Y*, and the remaining transcription factors acted as the predictors. We used the plsregress option from MATLAB R2010. The data was mean centered and variance scaled. Matlab uses the SIMPS algorithm to perform the regression on the data. The whole analysis results in a $BETA$vector of coefficients which describes the following relation between $Y$and $X$:

$$Y=X\times BETA+\mathrm{Re}siduals$$

Note that the vector $BETA$ is a $(n+1)$dimensional vector with the first entry as the intercept and the remaining $n$ entries as the coefficients for each of the remaining TF. The quality of the regression was calculated using using an R^2^ coefficient given by$R^{2}=1-\frac{\sum_{i=1}^{t} (\mathrm{Re}sidual_{i})^{2}}{\sum_{i=1}^{t} (Y_{i}-Y_{mean})^{2}}$. The total number of time points ($t$) including the undifferentiated stage are 4. For the PLSR plot in Fig 7, the R^2^ values were above 0.995.

Hierarchical clustering

We used hierarchical clustering on the entire dataset to identify the relationship between all the TFs and the treatments. Hierarchical clustering partitions the data into clusters through an iterative process, where similarity or dissimilarity between every pair of variables in the data matrix is calculated using an appropriate distance measure followed by grouping of the variables in close proximity using a linkage function. The clustering is performed in each of the dimension separately (genes and differentiation stages). We used the in-built Matlab functions *pdist* and *linkage* to perform the analysis on the mean expression data and the results were represented as a clustergram. We also tested the tree generated using different linkage measures and found all the trees to be similar with the cophenetic correlation coefficient greater than 0.9.
