# Supplementary material for: Potential for Pancreatic Maturation of Differentiating Human Embryonic Stem Cells Is Sensitive to the Specific Pathway of Definitive Endoderm Commitment
Source: PLoS One. 2014 Apr 17;9(4):e94307. doi: 10.1371/journal.pone.0094307 (PMC3990550; doi:10.1371/journal.pone.0094307)
Supplement: Table S1 — Media formulations for all stages of differentiation. (DOCX) [file pone.0094307.s005.docx]

**Media formulation for all stages of differentiation**

| **Stage** | **Base Media** | **Supplements** | **Inducers** | **Comments** |
| --- | --- | --- | --- | --- |
| **Definitive Endoderm** | DMEM/F12 | 0.2% BSA | 100ng/ml Activin A | Day 0-4 |
|  |  | B27 | *100ng/ml Fgf2 |  |
|  |  |  | *100ng/ml Bmp4 |  |
|  |  |  | *25ng/ml Wnt3a |  |
|  |  |  | *1μM Wortmannin |  |
| **Pancreatic Progenitor** | DMEM/F12 | 0.2% BSA | 0.2µM KAAD-Cyclopamine | Day 4-8 |
|  |  | B27 | 2µM All-Trans Retinoic Acid | Day 6-8 |
| **Maturation** | DMEM/F12 | 0.2% BSA | 10mM Nicotinamide | Day 8-End |
|  |  | B27 | 30μM DAPT | Day 10-End |
|  |  | 25 μg/ml Insulin |  |  |
|  |  | 50 μg/ml Transferrin |  |  |
|  |  | 30nM Sodium Selenite |  |  |
|  |  |  |  |  |
| ** Either/or |  |  |  |  |
