# Supplementary material for: Potential for Pancreatic Maturation of Differentiating Human Embryonic Stem Cells Is Sensitive to the Specific Pathway of Definitive Endoderm Commitment
Source: PLoS One. 2014 Apr 17;9(4):e94307. doi: 10.1371/journal.pone.0094307 (PMC3990550; doi:10.1371/journal.pone.0094307)
Supplement: Table S2 — Primers for quantitative RT-PCR. (DOCX) [file pone.0094307.s006.docx]

**Primers for qPCR**

| **Gene** | **Primers** | **Reference** |
| --- | --- | --- |
| GAPDH | ACGACCACTTTGTCAAGCTCATTTC | D'Amour et al., 2006 |
|  | GCAGTGAGGGTCTCTCTCTTCCTCT |  |
| SOX17 | CTCTGCCTCCTCCACGAA | Osafune et al., 2008 |
|  | CAGAATCCAGACCTGCACAA |  |
| CER | ACAGTGCCCTTCAGCCAGACT | D'Amour et al., 2006 |
|  | ACAACTACTTTTTCACAGCCTTCGT |  |
| FOXA2 (Hnf3b) | GGAGCGGTGAAGATGGAA | Osafune et al., 2008 |
|  | TACGTGTTCATGCCGTTCAT |  |
| CXCR4 | CACCGCATCTGGAGAACCA | D'Amour et al., 2006 |
|  | GCCCATTTCCTCGGTGTAGTT |  |
| PDX1 | AAGTCTACCAAAGCTCACGCG | Kroon et al., 2008 |
|  | GTAGGCGCCGCCTGC |  |
| PTF1 | GAAGGTCATCATCTGCCATCG | D'Amour et al., 2006 |
|  | GGCCATAATCAGGGTCGCT |  |
| NGN3 | CCTTACCCTTAGCACCA | Kroon et al., 2008 |
|  | CCCTCTACTCCCCAGTCTCC |  |
| HNF6 | TGTGGAAGTGGCTGCAGGA | Zhang et al., 2009 |
|  | TGTGAAGACCAACCTGGGCT |  |
| HLXB9 | CACCGCGGGCATGATC | D'Amour et al., 2006 |
|  | ACTTCCCCAGGAGGTTCGA |  |
| PAX4 | TCTCCTCCATCAACCGAGTC | Zhang et al., 2009 |
|  | GAGCCACTATGGGGAGTGAG |  |
| PAX6 | CGAATTCTGCAGGTGTCCAA | Zhang et al., 2009 |
|  | ACAGACCCCCTCGGACAGTAAT |  |
| NKX6-1 | AGACCCACTTTTTCCGGACA | Zhang et al., 2009 |
|  | CCAACGAATAGGCCAAACGA |  |
| NKX2-2 | GGCCTTCAGTACTCCCTGCA | D'Amour et al., 2006 |
|  | GGGACTTGGAGCTTGAGTCCT |  |
| ISL1 | GATCTATGTCACCTCGCAAGG | Osafune et al., 2008 |
|  | TACAACCACCATTTCACTG |  |
| MAFA | CTTCAGCAAGGAGGAGGTCATC | Zhang et al., 2009 |
|  | CTCGTATTTCTCCTTGTACAGGTCC |  |
| SOMATOSTATIN | CCCAGACTCCGTCAGTTTCT | Zhang et al., 2009 |
|  | ATCATTCTCCGTCTGGTTGG |  |
| GLUT2 | AGCTTTGCAGTTGGTGGAAT | Hui et al., 2001 |
|  | AATAAGAATGCCCGTGACGA |  |
| INSULIN | AAGAGGCCATCAAGCAGATCA | D'Amour et al., 2006 |
|  | CAGGAGGCGCATCCACA |  |
| GLUCAGON | AGGCAGACCCACTCAGTGA | Osafune et al., 2008 |
|  | AACAATGGCGACCTCTTCTG |  |
