# Supplementary material for: Potential for Pancreatic Maturation of Differentiating Human Embryonic Stem Cells Is Sensitive to the Specific Pathway of Definitive Endoderm Commitment
Source: PLoS One. 2014 Apr 17;9(4):e94307. doi: 10.1371/journal.pone.0094307 (PMC3990550; doi:10.1371/journal.pone.0094307)
Supplement: Table S3 — Antibodies for flow cytometry and immunocytochemistry. (DOCX) [file pone.0094307.s007.docx]

**Antibodies for FC and ICC**

| **Antibody** | **Source** | **Application** | **Dilution** |
| --- | --- | --- | --- |
| FOXA2 | Santa Cruz Biotechnologies sc-20692 | Flow | 1:500 |
| C-Peptide | Abcam ab14181 | Flow/ICC | 1:1000/1:500 |
| Alexafluor 488 or 647 | Invitrogen | Flow/ICC | 1:1000/1:500 |
| SOX17 | Santa Cruz Biotechnologies sc-17355 | ICC | 1:200 |
| PDX1 | Santa Cruz Biotechnologies sc-14662, 1:200 | ICC | 1:200 |
| Hoescht stain | Invitrogen | ICC | 1:1000 |
